# Real‐world efficacy and potential mechanism of resistance of icotinib in Asian advanced non‐small cell lung cancer with EGFR uncommon mutations: A multi‐center study

**DOI:** 10.1002/cam4.2652

**Published:** 2019-11-06

**Authors:** Lei Lei, Wen‐xian Wang, You‐cai Zhu, Jin‐luan Li, Yong Fang, Hong Wang, Wu Zhuang, Yin‐bin Zhang, Li‐ping Wang, Mei‐yu Fang, Chun‐wei Xu, Xiao‐jia Wang, Tang‐feng Lv, Yong Song

**Affiliations:** ^1^ Department of Medical Oncology Cancer Hospital of University of Chinese Academy of Sciences (Zhejiang Cancer Hospital) Hangzhou Zhejiang People's Republic of China; ^2^ Department of Thoracic Disease Center Zhejiang Rongjun Hospital Jiaxing Zhejiang People's Republic of China; ^3^ Department of Radiotherapy Xiamen Cancer Hospital The First Affiliated Hospital of Xiamen University Xiamen Fujian People's Republic of China; ^4^ Department of Oncology Sir Run Run Shaw Hospital Hangzhou Zhejiang People's Republic of China; ^5^ Department of Lung Cancer The Fifth Medical Center General of PLA Beijing People's Republic of China; ^6^ Department of Medical Oncology Fujian Provincial Cancer Hospital Fujian Medical University Cancer Hospital Fuzhou Fujian People's Republic of China; ^7^ Department of Oncology The Second Affiliated Hospital of Medical College Xi′an Jiaotong University Xi'an Shaanxi People's Republic of China; ^8^ Department of Oncology Baotou Cancer Hospital Baotou Inner Mongolia People's Republic of China; ^9^ Department of Pathology Fujian Cancer Hospital Fujian Medical University Cancer Hospital Fuzhou Fujian People's Republic of China; ^10^ Department of Respiratory Medicine Jinling Hospital Nanjing Jiangsu People's Republic of China

**Keywords:** ctDNA, *EGFR*, icotinib, NGS, NSCLC

## Abstract

The response to icotinib in advanced non‐small cell lung cancers (NSCLC) with *EGFR* uncommon mutation (*EGFR*um) is unclear. Here we reported the efficacy and potential resistance mechanism of icotinib in Chinese *EGFR*um NSCLC patients. Between July 2013 and November 2016, 3117 NSCLC patients were screened for *EGFR*um in a multi‐center study in China. Circulating tumor DNA (ctDNA) was detected and analyzed using next‐generation sequencing (NGS) after progression from icotinib. The efficacy, safety and the potential resistance mechanism of icotinib were explored. After a median follow‐up of 6.2 months, 69 patients (70.41%) developed disease progression, the objective rate (ORR) and disease control rate (DCR) were 13.27% and 29.59% respectively, and the median progression‐free survival (PFS) was 5.5 months (95% CI: 1.2‐13.0 months). Both complex‐pattern with *EGFR* classical mutations (*EGFR*cm) and single‐pattern have better PFS than complex‐pattern without *EGFR*cm (median PFS was 7.2 (95% CI: 4.65‐9.75), 5.2 (95% CI: 3.24‐7.16) and 3.2 (95% CI: 2.97‐3.44) months, respectively, *P* < .05); patients harboring S768I mutation had the worst PFS than others (2.0 months, *P* < .05). Diarrhea was the most frequent side effect (42.9%). Forty‐eight (69.6%) patients developed drug resistance after 3.0 months and 81.2% of them acquired T790M mutation. Better response was observed in complex‐pattern with the *EGFR*cm group. S768I mutation carriers may not benefit from icotinib. Acquired T790M mutation was common in icotinib‐resistant *EGFR*um NSCLC patients.

## Graphical abstract

Better response to icotinib was observed in complex‐pattern with EGFRcm group. S768I mutation carriers may not benefit from icotinib. Acquired T790M mutation was common in icotinib‐resistant EGFRum NSCLC patients.

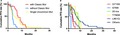

## 1 INTRODUCTION

At present, lung cancer still has the highest incidence and mortality in all cancers worldwide, and non‐small cell lung cancer (NSCLC) accounts for 80%‐85% of all lung cancer.^1^ Some patients have advanced stage lung cancer when initially diagnosed. The traditional therapy for the advanced stage NSCLC is mainly systemic chemotherapy.^1^ After the epidermal growth factor receptor (*EGFR*) gene mutation‐driven NSCLC has been claimed, tyrosine kinase inhibitors (TKIs) have already replaced traditional chemotherapy as the standard first‐line therapy for advanced‐stage NSCLC patients with *EGFR*‐sensitive mutations.^2^, ^3^ Unlike those NSCLC patients with classical *EGFR* mutations (*EGFR*cm),^2^, ^4^, ^5^ the prevalence and TKIs response in *EGFR* uncommon mutation (*EGFR*um) NSCLC patients remain under study.

Consistently in literature, about 10% of all *EGFR* mutation carriers are patients with *EGFR*um regardless of race.^5^, ^6^, ^7^ At present, *EGFR*um could be divided into three types, including point mutation or replication in 18‐21 exon, de novo T790M and 20 exon insertion (20ins) mutations.^8^ Research showed that patients with *EGFR*um are more common in complex mutant pattern and show less response to TKIs than *EGFR*cm.^9^, ^10^ Although afatinib, a second‐generation TKI, is recently recommended for some of *EGFR*um (G719X/S768I/L861Q) carriers, the first generation TKIs are still worth to be studied in this population including other *EGFR*ums.^11^, ^12^

Icotinib is a quinazoline derivative that reversibly binds to the ATP binding site of *EGFR* protein, thereby preventing lung cancer cells from completing the signal transduction cascade and stopping the cell from overproliferating.^13^ It was developed and confirmed efficacy as the first‐generation TKIs in a registered clinical trial in China.^14^ Here, we have presented the clinical response and genetic profiling of resistance to icotinib in advanced NSCLC patients with *EGFR*um from a retrospective study in China.

## 2 METHODS

### 2.1 Patient selection

**Figure 1:**
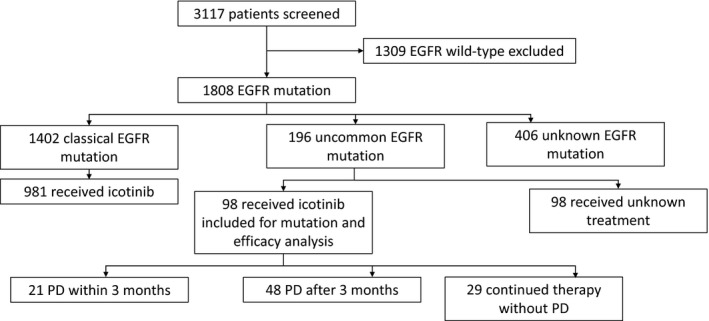
Flow chart of study populations

The medical and *EGFR* genotype data of 3117 NSCLC patients were retrospectively collected from multicancer centers in China between July 2013 and November 2016 (Figure 1). Ninety‐eight *EGFR*um patients treated with icotinib (125 mg, tid) were enrolled for analysis. Complex *EGFR* mutation was defined as the coexistence of two different *EGFR* mutation spots. All participants gave written the informed consent and the project was approved by the hospitals′ ethics committee. Medical record data on the histology and staging of all patients have been reconfirmed by two pathologists at initial diagnosis. The follow‐up data were collected until patients developed disease progression or death. Previous TKIs treated, unmeasurable lesions or less than 3 months of life expectancy were the key exclusions.

### 2.2 Targeted next‐generation sequencing

Genomic DNA sequencing libraries were prepared using the protocols recommended in the Illumina TruSeq DNA Library Preparation Kit. For samples close to the minimum input requirement, additional precapture PCR cycles were performed to generate sufficient PCR product for hybridization. The libraries were hybridized to custom‐designed probes (Integrated DNA Technology) including all exons of 170 genes and selected intron of *ALK*, *RET* and *ROS1* for the detection of genomic rearrangements. DNA sequencing was performed on a HiSeq3000 sequencing system (Illumina, San Diego, CA) with 2 × 75 bp paired‐end reads. The reads were aligned to the human genome build GRCh37 using BWA (a Burrows‐Wheeler aligner). Somatic single nucleotide variant (sSNV) and indel calls were generated using MuTect and GATK, respectively. Somatic copy number alterations were identified with CONTRA. Genomic rearrangements were identified by the software developed in‐house analyzing chimeric read pairs.

### 2.3 Efficacy evaluation and follow‐up

Routine enhanced computed tomography (CT) scans were performed for baseline measurement and evaluation of response to icotinib. The period of follow‐up assessment was every 3.0 months after taking icotinib until the trigger of disease progression or death. Two proficient radiologists independently confirmed the efficacy of treatment based on the Response Evaluation Criteria in Solid Tumors (RECIST).^15^ Progression‐free survival (PFS) was defined as the interval between the start of icotinib treatment to the last follow‐up, disease progression or death from any cause which came first.

### 2.4 Toxicity evaluation

The side effects profile of icotinib and the cause of death were collected from the medical records. The severity of adverse events was evaluated according to the National Cancer Institute Common Toxicity Criteria version 4.0 (CTC4.0).^16^ Dose reduction or stop‐using of icotinib due to severe toxicity have been followed by local guidelines. No patients has developed death by any cause during the follow‐up period.

### 2.5 Statistical analysis

Clinical and mutational characteristic data were analyzed using SPSS software (Version 22.0, SPSS Inc). Categorical variables were compared between the *EGFR* mutant subgroups using Chi‐square (*χ* ^2^) and Fisher's exact tests. PFS rates were estimated using the Kaplan‐Meier method and examined using the log‐rank test. Multivariable analysis was assessed using the Cox proportional hazards model for PFS rate. The age of diagnosis, smoking status, tumor stage and Eastern Cooperative Oncology Group (ECOG) Performance Status (PS) score were adjusted. Differences were confirmed by two‐sided *P* < .05.

## 3 RESULTS

### 3.1 Clinicopathologic characteristics of icotinib treated NSCLC patients with EGFRum

About 10.88% of *EGFR*um patients were identified from multi‐cancer centers in China between July 2013 and November 2016 and half of them accepted icotinib treatment. *EGFR*um patients were diagnosed at younger age (65 y.o. as cutoff, *χ* ^2^ = 14.32, *P* < .001) and more frequent adenocarcinoma histology (*χ* ^2^ = 20.92, *P* < .001) than *EGFR*cm patients. No significant difference in gender, smoking history and ECOG PS was observed. *EGFR*um patients were primarily administered icotinib after second‐line treatment (78/98, 79.6%) (Table 1).

**Table 1:** Baseline characteristics in 98 EGFR uncommon mutation NSCLC patients

Characteristic	N = 98 (%)
Median age (y)	
<65	68 (69.4)
≥65	30 (30.6)
Sex	
Male	53 (54.1)
Female	45 (45.9)
Smoking status	
Present or former smoker	38 (38.8)
Nonsmoker	60 (61.2)
ECOG PS	
0‐1	70 (71.4)
2‐3	28 (28.6)
Histology	
Adenocarcinoma	91 (92.9)
Nonadenocarcinoma	7 (7.1)
Treatment lines	
First	3 (3.1)
Second	17 (17.3)
Third	78 (79.6)

Abbreviations: ECOG PS, Eastern Cooperative Oncology Group performance status; EGFR, epidermal growth factor receptor; NSCLC, non‐small cell lung cancer.

### 3.2 Efficacy of icotinib in EGFRum patients

**Figure 2:**
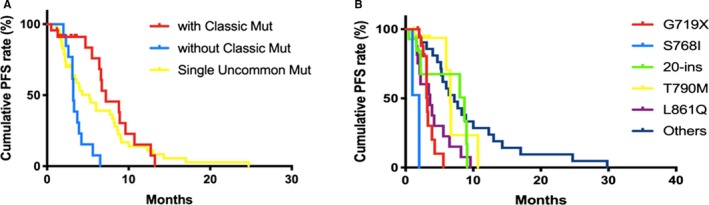
Comparisons of PFS rate in *EGFR*um patients by mutation patterns (A) and point mutations (B). A, Complex‐pattern without *EGFR* classic mutant (blue) carriers have worse PFS than single‐pattern (yellow) and complex‐pattern with *EGFR* classic mutant(red) (*P* < .05). B, S768I mutation carriers (blue) have the worst PFS than others (2.0 months, *P* < .05). *EGFR*um, epidermal growth factor receptor uncommon mutation; PFS, progression‐free survival

The median follow‐up time was 6.2 months, 70.41% (69/98) developed disease progression with an objective response rate (ORR) of 13.27% and a disease control rate (DCR) of 29.59%. No patient with complete response (CR) was observed (Table 2). The median PFS was 5.5 months (0.5‐29.8, 95% confidence interval (CI): 1.2‐13.2 months) in the whole group. The PFS of complex‐pattern without *EGFR*cm was significantly shorter than that of mutant patterns (3.2 months, *P* < .05). The S768I mutant group had the worst PFS compared to others (2.0 months, *P* < .001) (Figure 2).

**Table 2:** Efficacy of icotinib in EGFR uncommon mutation NSCLC patients

Contained mutation	CR (n)	PR (n)	SD (n)	PD (n)	Total	ORR (%)	DCR (%)
Exon 18	0	3	4	17	24	12.50	29.17
Exon 20	0	9	10	38	57	15.79	33.33
Exon 21	0	1	2	21	24	4.17	12.50
& others	0	0	0	8	8	0	0
Total	0	13	16	69	98	13.27	29.59

Abbreviations: CR, complete response; DCR, disease control rate; EGFR, epidermal growth factor receptor; NSCLC, non‐small cell lung cancer; ORR, objective response rate; PD, progressive disease; PR, partial response; SD, stable disease.

### 3.3 Side effects and tolerance of icotinib in EGFRum patients

The common side effects of icotinib included diarrhea (42.9%), elevated aminotransferase (28.6%), abdominal pain and constipation (9.2%), oral cavity mucous membrane inflammation (8.2%), nausea and vomiting (7.1%). Any grade 3/4 adverse events (AEs) and the dose reduction induced by AEs or temporary discontinuation of treatment have been reported in five cases (5.1%) and in three cases (3.1%), respectively (Table 3).

**Table 3:** Adverse events of icotinib in 98 EGFR uncommon mutation NSCLC patients

Symptoms	Any grade	No. (%) of AE Grade 3/4	Dose‐adjustment/interruption
Diarrhea	42 (42.9)	2	2
Nausea and vomit	7 (7.1)	1	1
Abdominal pain and constipation	9 (9.2)	1	0
Alanine aminotransferase elevation	28 (28.6)	1	0
Elevated bilirubin	5 (5.1)	0	0
Neutropenia	3 (3.1)	0	0
Anemia	2 (2.0)	0	0
Malaise	2 (2.0)	0	0
Back pain	1 (1.0)	0	0
Numbness/abnormal feeling	1 (1.0)	0	0
Fever	1 (1.0)	0	0
Oral mucositis	8 (8.2)	0	0
Rash/ Pruritus	6 (6.1)	0	0
Others	4 (4.1)	0	0
Summary	119	5	3

Abbreviations: AE, adverse event; EGFR, epidermal growth factor receptor; NSCLC, non‐small cell lung cancer.

### 3.4 Genetic profiling of 48 EGFRum patients who developed resistance to icotinib

**Figure 3:**
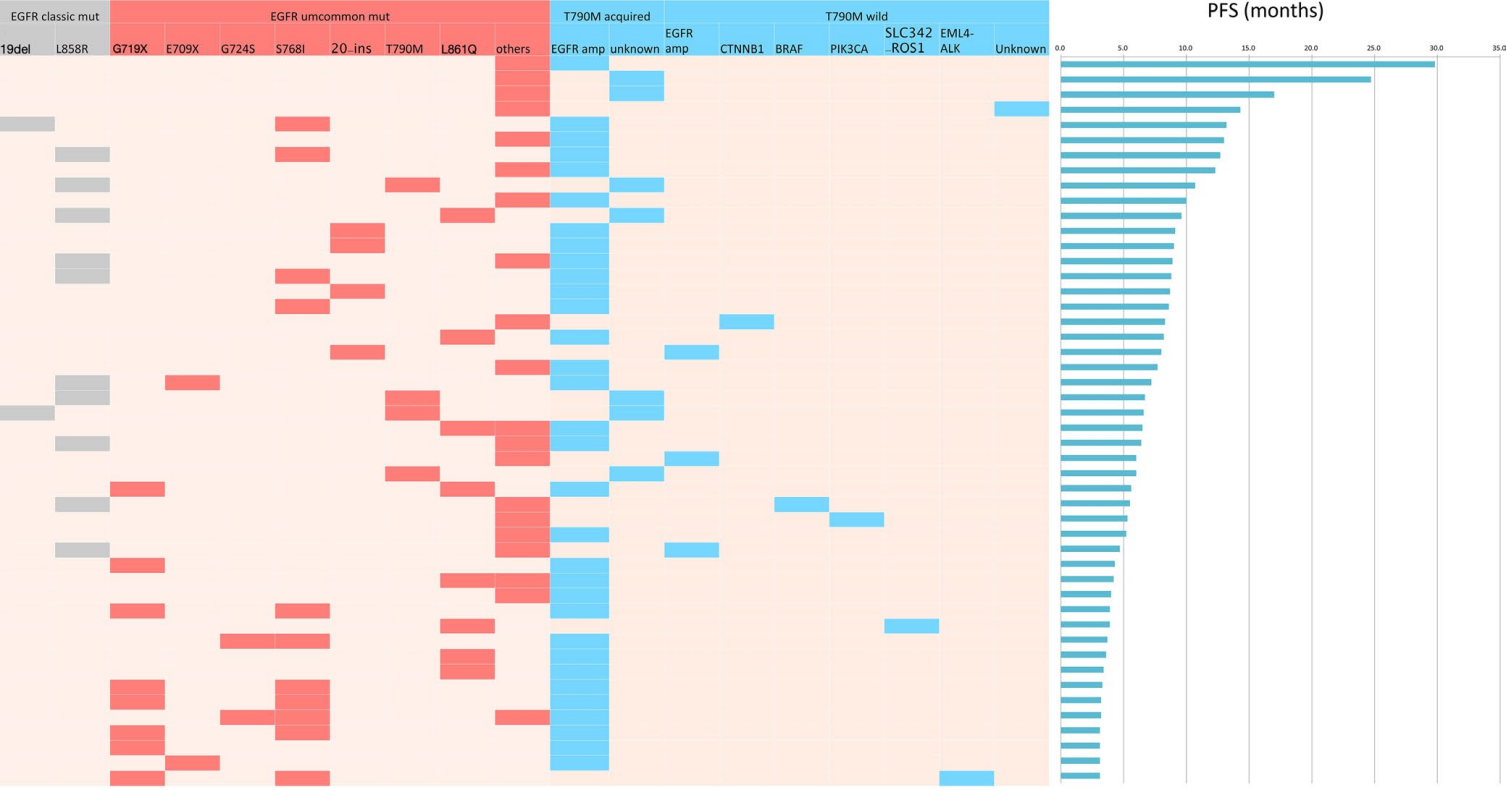
Plasma circulating tumor DNA sequencing results for 48 *EGFR*um NSCLC patients with acquired drug resistance. The heat map showed the baseline *EGFR*um patterns (gray and red), genetic profiling after disease progression (blue) and PFS at the very right side. *EGFR*um, epidermal growth factor receptor uncommon mutation; NSCLC, non‐small cell lung cancer; PFS, progression‐free survival.

We divided 48 EGFRum patients who developed resistance to icotinib into two groups, T790M acquired and T790M wild types. A total of 81.2% (39/48) patients harbored T790M acquired mutation, 82% (32/39) of them accompanied by *EGFR* amplification at the same time. In the T790M wild type group, Three patients 33.3%(3/9) harbored *EGFR* amplification, five patients harbored *CTNNB1*, *PIK3CA*, *BRAF, EML4‐ALK*, and *SLC342‐ROS1*, respectively. One patient (11.1%, 1/9) harbored unknown mutation (Figure 3). No significant difference in median PFS has been observed in the group of patients with T790M acquired mutation (6.6 vs 5.3 months, *χ* ^2^ = 0.58, *P* = .45).

## 4 DISCUSSION

In this study, we retrospectively explored the efficacy and resistance mechanism of icotinib in advanced *EGFR*um NSCLC patients from a multi‐center in China. About 10% *EGFR*ums were found and the frequency was consistent with literature.^17^, ^18^ After a median follow‐up of 6.2 months, 69 patients (70.41%) developed disease progression and the median PFS was 5.5 months (95% CI: 1.2‐13.2 months). The ORR and DCR were 13.27% and 29.59%, respectively. Diarrhea was the most common AE but was manageable.

Up to 24% *EGFR*um NSCLC patients occurrent as complex mutation pattern from a large cohort study with genome‐wide sequencing data.^9^ The complex‐pattern with *EGFR*cm accounted for 23.5% (23/98) in our study and was associated with significantly better PFS than those without *EGFR*cm. The outcome of complex‐pattern with *EGFR*um is complicated and associated with different compositions of mutations.^17^, ^19^ However, one study found no significant difference of outcome under TKI treatment between complex and single‐pattern of *EGFR*um patients.^20^ In this study, most complex‐pattern of mutations (20/22) included *EGFR*cm which could no doubt have positive impact on the conclusion. Because of the predominant *EGFR*cm composition in that study, the non‐significant conclusion could be biased.

S768I mutation accounts for about 0.49% of all *EGFR* mutations and often appears as complex‐pattern with other mutations.^21^ About 26.5% of *EGFR*um patients in our study haboring S768I mutation and the frequency was pretty similar with results from other studies.^22^, ^23^ Notably, we found that patients who harbored an S768I mutation had the worst PFS compared with G719X, L861Q, 20‐ins, and de novo T790M mutation carriers. Studies have shown that the S768I mutation may be associated with drug resistance to the first generation of *EGFR*‐TKI drugs.^24^ A Ba/F3 cell line resistance experiment showed that the second‐generation *EGFR*‐TKI (afatinib) was more effective than the first (erlotinib) and third (osimertinib) generation EGFR‐TKIs. The IC_50_ concentrations of the three are 0.7, 146 and 49 nmol/L.^25^ However, S768I carriers could possibly have partial sensitivity to the first generation of *EGFR*‐TKIs.^26^ The efficacy of *EGFR*‐TKIs in S768I carriers remains to be determined.^27^, ^28^

We found T790M acquired mutation was the dominant acquired genetic mutation when patients with *EGFR*um developed resistance to icotinib, meanwhile, T790M mutation always co‐occurrence with *EGFR* amplification. Acquired T790M mutation after progression from TKIs was found as a positive prognostic factor compared to the wild type in *EGFR*cm NSCLC patients.^28^, ^29^ We did found a similar tendency in *EGFR*um patients but failed to reach the statistical difference. Of note, there were two patients acquired T790M acquired mutation harvested more than two years of PFS. One patient harboring *EGFR*‐KDD (*EGFR* Kinase Domain Duplication) harvested the longest PFS which was up to almost 30 months. *EGFR*‐KDD belongs to a structural alteration but not point mutation in the *EGFR* gene, which could be a biomarker for TKIs sensitivity prediction.^30^ The second patient harboring the *EGFR*‐SEPT14 fusion, another structural alteration in the *EGFR* gene, reached long PFS as 24.7 months.^31^

However, there are some shortcomings in our study. Firstly, it is a retrospective study and the selection bias could not be neglected. The frequency of *EGFR*um is very low which means a large cohort for screening needs to be well‐prepared before research. Secondly, icotinib is not the standard TKIs recommendation in many other countries and it could also be assumed to be inactive in NSCLC patients with *EGFR*um. However, icotinib has been widely used in China and insurance covered, especially in those rural areas. Due to the complicated outcomes and lack of target therapy in advanced NSCLC patients with *EGFR*um, searching for actionable molecular targets by illuminating the resistance mechanism of TKIs would be meaningful in future study.

In summary, better response was observed in complex‐pattern with *EGFR*cm in our study. S768I mutation carriers may not benefit from icotinib. Acquired T790M mutation may be the genetic feature of icotinib resistant in advanced NSCLC with *EGFR*um patients.

## CONFLICT OF INTEREST

None of the authors has any conflict of interest.
